# Tunable lasing in cholesteric liquid crystal elastomers with accurate measurements of strain

**DOI:** 10.1038/srep17739

**Published:** 2015-12-04

**Authors:** Andrii Varanytsia, Hama Nagai, Kenji Urayama, Peter Palffy-Muhoray

**Affiliations:** 1 Liquid Crystal Institute and Chemical Physics Interdisciplinary Program, Kent State University, Kent, Ohio 44242, USA; 2 Department of Macromolecular Science & Engineering, Kyoto Institute of Technology, Sakyo-ku, Kyoto 606-8585, Japan

## Abstract

We report wide range and reversible tuning of the selective reflection band of a single crystal cholesteric liquid crystal elastomer (CLCE). The tuning is the result of mechanical shortening of the helical pitch achieved by imposing a uniform uniaxial strain along the helical axis. On doping the CLCE sample with a laser dye, we observe lasing from the CLCE in both glassy and rubbery states. By changing the cholesteric pitch, mechanical compression provides tuning of the laser emission from the dye doped CLCE over a significant part of the fluorescence band of the laser dye. In this work we demonstrate for the first time that both the CLCE pitch and the lasing wavelength are linearly dependent on the strain imposed on the CLCE film.

Liquid crystals (LC) self-assemble into variety of mesophases with spatially periodic molecular arrangements. These are characterized by orientational order, and varying amounts of positional order. The orientational order can often be characterized by a scalar order parameter, which gives a measure of the degree of oreintational order, and a unit vector, called the director, which indicates the direction of alignment. Supramolecular periodicity together with the anisotropic properties of LC molecules results in spontaneously formed photonic crystal with a band-gap where the propagation of electromagnetic waves with wavelengths comparable to the period of these structures is forbidden^1^ for certain eigenmodes. Cholesteric liquid crystals (CLC) spontaneously form a supramolecular helical structures with the cholesteric pitch corresponding to continuous rotation of a local director through an angle of 2π. The helical structure of CLCs provides a periodic modulation of the dielectric tensor along helical axis creating a 1D photonic crystal with a strong Bragg reflection of light with the same handedness as the CLC helix^2^.

CLCs with a selective reflection band were predicted to serve as a distributed feedback cavity^3^ and demonstrated mirrorless distributed feedback lasing^4^,^5^,^6^. In order to achieve lasing, CLC film needs to be optically pumped using an external light source and its constituents need to have fluorescent emission within the selective reflection band. Due to the band structure, the fluorescent emission is suppressed in the reflection band and is enhanced at the band edges. This provides gain for bidirectional lasing along the helical axis of CLC^2^,^6^,^7^. Although, some liquid crystal materials have strong enough fluorescence to produce distributed feedback lasing^8^ usually a small amount of fluorescent laser dye with desired excitation and emission bands is additionally introduced into a CLC mixture. Distributed feedback lasing has been observed in a large variety of LC phases and materials: in pure^8^, dye doped^4^,^5^,^6^, and using Forster energy transfer between two dyes in low molecular weight СLCs^9^, in three-dimensional photonic crystal of the liquid crystal blue phase^10^, ferroelectric liquid crystals^11^,^12^, free-standing polymer CLC films^13^,^14^, solid polymer CLC films based on cellulose^15^, deformable rubbery CLC elastomers^16^,^17^,^18^ and multilayered films with CLCE serving as tunable mirrors^19^,^20^. Laser emission has been demonstrated from different CLC materials in a broad spectral range from near UV to near IR; the soft matter aspect of such lasers enabled the tuning of the lasing wavelength using a variety of techniques^2^,^7^.

CLCEs are soft solids formed by mesogenic molecules incorporated into a polymer network with helical structure similar to low molecular weight CLCs. The interaction between the polymer chains and mesogens couples orientational order to mechanical strain. Such a coupling allows a variety of electro-mechanical and opto-mechanical effects with a great potential for applications^21^. Tunable lasing from single crystal cholesteric liquid crystal elastomer was observed for the first time and reported in 2001^16^. It was shown that extensive mechanical deformation of the elastomer film shortens CLC pitch creating a dramatic blue-shift of the selective reflection band and therefore of the wavelength of laser emission. The pitch of the CLCE was changed by a biaxial strain normal to the helical axis. Such a deformation effectively shortens helical pitch, leaving the helical structure essentially unchanged, but creating a nontrivial deformation with additional shear near the sample edges. As result, helical pitch is not uniform across the CLCE film, and it accurate determination is challenging.

An alternative method of mechanical tuning of the lasing wavelength from single^18^ or layered^19^,^20^ CLCEs was demonstrated by imposing a uniaxial extension on the CLCE film in the direction orthogonal to helical axis. Uniaxial elongation perpendicular to helical axis of CLCE creates an extension of the material along the direction of strain and a contraction with nonlinear power law along the two orthogonal directions. This type of deformation of CLCEs has been extensively discussed theoretically^22^,^23^,^24^,^25^ and observed experimentally^19^,^26^,^27^. The CLC director gradually reorients along direction of applied strain significantly deforming the CLC helix. Complicated compression and deformation of the CLC helix, caused by the uniaxial strain, follow with further elongation. Sufficiently large elongation is predicted and is experimentally observed to unwind the helical texture of the CLCE to produce a non-helical state.

In this work, we use an improved CLCE material and a new geometry for its deformation, different from that reported in our earlier paper^6^ well as from other papers on the topic of dye-doped lasing from CLCEs. We deform the CLCE film by imposing a uniaxial compression parallel to the helical axis when the sample is confined between two glass substrates. With such a mechanical system we are able to create an essentially uniform uniaxial strain in the whole area of the CLCE film and at the same time avoid complicated and non-uniform deformations of CLC helix or strain induced unwinding of CLCE helical structure. As result, we are able to accurately measure the shift of the selective reflection band and wavelength of laser emission as a function of the strain in the CLCE film.

## Synthesis and manufacturing of the sample

**Figure 1:**
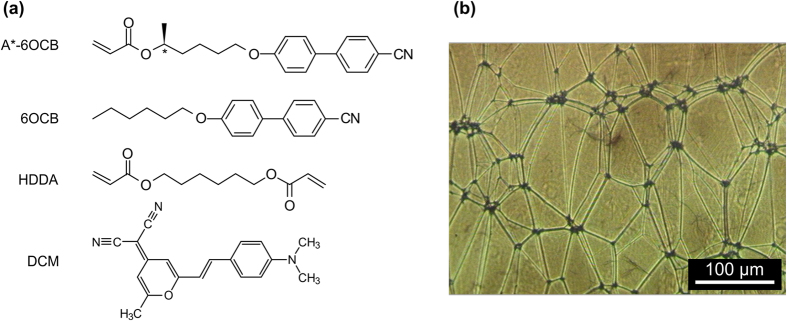
(**a**) Chemical structures of compounds, (**b**) POM images of the CLCE film.

The CLCE film was fabricated using the method described in detail elsewhere^28^. The photopolymerization of the reactive chiral monoacrylate (A*-6OCB; Fig. 1(a) ) and the diacrylate cross-linker (HDDA; Fig. 1(a)) was conducted in the presence of a non-reactive achiral nematic solvent (6OCB; Fig. 1 (a)) using IRGACURE® 784 as a photoinitiator. The mixture with a molar ratio of A*-6OCB, 6OCB, and HDDA of 1:0.82:0.07 was loaded in a 32 μm-thick glass cell whose surfaces were coated with rubbed polyimide layer, inducing planar alignment. The cell was left at 10 °C for 1 day so that the LC mixture could form a monodomain Grandjean cholesteric texture. After the irradiation with light at a wavelength of 526 nm for 30 min, the glass substrates were removed from the cell, and the resultant gel film was allowed to swell in dichloromethane to wash away the unreacted materials and 6OCB. The swollen gel was gradually deswollen by adding methanol to the swelling solvent, resulting in a fully dried film. The thickness of the resulting CLCE film was 25 μm. Fig. 1(b) shows transmission mode polarizing optical microscope (POM) image of the CLCE film with visible defect lines equivalent to oily streaks observed in low molecular weight CLCs with good planar alignment.

We used the laser dye 4-(dicyanomethylene)-2-methyl-6-(4-dimethylamino styryl)-4-H-pyran (DCM) (from Exciton, Inc.) to achieve lasing, Fig. 1(a). The laser dye was introduced into the sample by swelling the CLCE in its glassy state at room temperature in a toluene followed by a contraction in methanol. The sample was swollen in a saturated solution of DCM in toluene for ~15 minutes. Following this, the toluene solution with DCM was slowly replaced by a saturated solution of DCM in methanol. Solvent replacement was performed in steps; by adding a small amount of methanol solution into the vial containing the swollen sample with subsequent gentle mixing and removal of a similar amount of the resulting solution of both solvents. The solvent replacement procedure was repeated until only a very small amount of toluene was left in the vial and the sample was completely contracted. Slow contraction of the sample by gradual replacement of solvents is critical to prevent a rapid decrease of volume which can result in cracking or breaking of the CLCE film. The concentration of the dye in the CLCE film after swelling is ~0.02 wt.%.

## Experimental results

**Figure 2:**
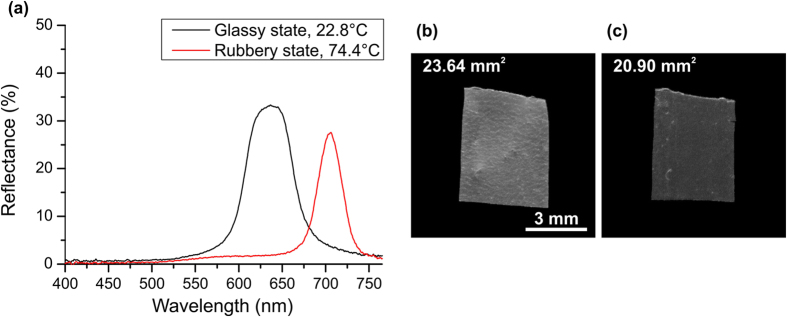
Reflectance from an undeformed CLCE film (a) and corresponding photographs with measured film area in the glassy state at T = 22.8 °C (b) and in the rubbery state at T = 74.4 °C (c).

The CLCE film produced is initially in the ordered state with helical axis perpendicular to the surface of the film with a clear selective reflection band, as shown on Fig. 2(a). The selective reflection band of the CLCE film exists only for right-handed circularly polarized light and the film is nearly transparent for left-handed circularly polarized light. Reflectance of circularly polarized light was measured with an Ocean Optics UV-Vis U2000 spectrometer using film circular polarizers with extinction coefficient of 500:1.

Close to room temperature, at ~23 °C, the CLCE film is in a brittle glassy state. Appreciable mechanical deformation of the CLCE film is possible only in its rubbery state, above the glass transition temperature of *T*_g_ ~70 °C. Upon transition from the glassy to the rubbery state, the selective reflection band of the CLCE film narrows in width and its position red-shifts indicating the considerable temperature dependence of the refractive indices and of the helical pitch of the CLCE film. Reflectance spectra from an undeformed CLCE film in the glassy state near room temperature (22.8 °C) and from an undeformed CLCE film in the rubbery state at the high temperature of the experiment (74.4 °C) are shown on Fig. 2(a–c) together with corresponding photographs and measured area of the CLCE film. The narrower selective reflection band in the rubbery state indicates a smaller birefringence of the CLCE film than in the glassy state. The full width at half maximum (FWHM) of the selective reflection band of the CLCE film at 22.8 °C is 60.8 nm and at 74.4 °C it is 34.3 nm. The red-shift of the selective reflection band and decrease of its area is a direct evidence of an increase of the helical pitch and of the film thickness as the CLCE film is heated from the glassy to the rubbery state above the glass transition temperature Tr_g_.

**Figure 3:**
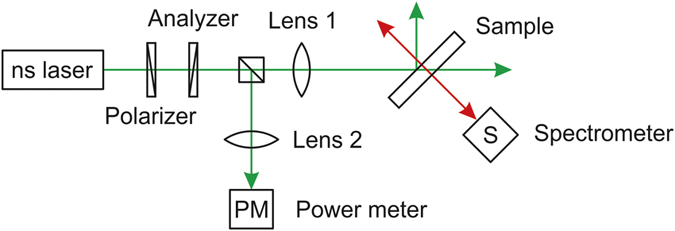
Experimental setup for the lasing experiment.

In order to achieve lasing, the sample was optically pumped using a frequency doubled Coherent Nd:YAG laser, with 7.5 ns pulses and rep. rate of 2 Hz at λ = 532 nm. The intensity of the pump beam was controlled with a polarizer and an analyzer. The pump beam was divided in two to allow simultaneous measurements of the pump energy and emission wavelength. The pump beam with 9 mm diameter was focused on the CLC film using a 12 cm focal length lens to a beam waist of ~50 μm. The angle of incidence of the pump beam on the sample was ~45°. The pump energy was measured using a Molectron Optimum 4001 energy meter and the emission spectrum from the sample was measured using a Jobin Yvon-Spex TRIAX 550 spectrometer. The optical setup for lasing is shown on Fig. 3.

Initially, we verified that the CLCE film can lase, and we measured the lasing threshold at room temperature in the glassy state of the CLCE sample. Subsequently, we performed lasing experiments and demonstrated mechanical tuning of the wavelength of the laser emission from the sample in the high temperature (~75 °C) rubbery phase.

**Figure 4:**
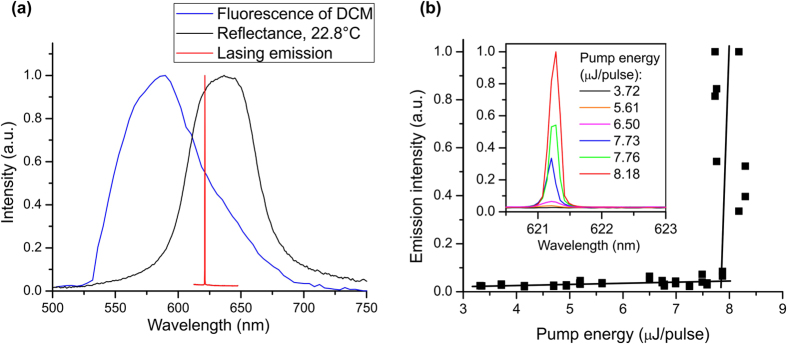
(**a**) Reflectance, fluorescence and lasing line observed with 532 nm pump (**b**) emission intensity from the CLCE sample as a function of pump energy and emission spectra at different pump energies.

When in the glassy state at room temperature (22.8 °C) the sample was supported by a single microscope glass slide. In order to create a weak adhesion to the CLCE film, the surface of microscope glass slide was coated with a thin film of silicone oil with viscosity of 1 m^2^/s. Lasing in the glassy state was observed closer to the high energy edge of the selective reflection band, as shown on Fig. 4(a). Although distributed feedback lasing from CLCs has lower threshold and at the low energy band edge^2^,^7^ and usually occurs there, in this particular case the fluorescence peak is better matched with the high energy band edge, and lasing was observed there. In the glassy state, lasing was observed within the wavelength range of 621.3 nm to 623.7 nm and occasionally more than one lasing line was visible. The simultaneous occurrence of lasing at slightly different wavelength may be explained by local structural heterogeneities produced by polymer network in highly crosslinked CLCEs comparing to low molecular weight CLCs^13^. In our CLCE film lasing lines with slightly different wavelengths were observed depending on the position of illuminated spot. The intensity of the emission from the sample was measured as a function of pump energy to determine the lasing threshold. As the pump power was increased gradually a clear lasing threshold could be observed at ~8 μJ/pulse, as shown on Fig. 4(b). The typical FWHM of lasing emission from the CLCE film was ~0.25 nm.

Pumping at high energies can cause thermal degradation of the distributed feedback cavity^29^ or burn the lasing spot of low molecular weight CLC sample. Similarly, when pumping with energies well above the lasing threshold, the CLCE film showed degradation of the lasing emission. Multiple sequential excitations of the same lasing spot gradually increased the lasing threshold, and eventually damaged the sample after which the lasing completely disappears. The damage threshold for our CLCE film in a glassy state at room temperature is approximately 65 μJ/pulse.

In the rubbery state above the glass transition temperature, the CLCE film is elastic and can support mechanical deformations such as stretching or compression. In our experiments, we uniformly compressed the CLCE film confined between two parallel glass plates. Such mechanical deformations were performed using a custom built device able to accurately control the separation of the glass plates using three screws with a fine (80 threads/inch) thread. Glass plates of 0.25 inch thickness were used to minimize bending. In order to prevent strong adhesion of the CLCE samples to the glass and possible damage to the film, again the glass plates were coated with a thin layer of silicone oil with viscosity of 1 m^2^/s. The compression cell with the enclosed CLCE film was located inside a plexiglass chamber with hot silicone oil of low viscosity (2 × 10^-3^ m^2^/s) pumped through it. The oil temperature and flow rate of were controlled by a Haake FE2 Heated Bath Circulator and adjusted to maintain the desired temperature of the compression cell. The temperature of the compression cell with the sample was measured using a calibrated thermistor next to the cell in the heating chamber. During all experiments with the CLCE in rubbery state, the sample temperature was maintained at 75 °C. The fluctuations of the temperature from uncontrolled heat losses were not more than +/–3 °C. Temperature fluctuations on this scale are not expected to affect significantly the optical and mechanical properties of the CLCE film.

Compression of the CLCE film in its rubbery state between parallel plates creates a uniaxial strain enlarging the area of compressed film. Since the strain is applied along the helical axis, due to the strong coupling between the CLCE director and polymer network the CLC pitch shortens with the macroscopic compression of the CLCE film. The pitch *p* of the CLC can be calculated from the known ordinary *n*_*o*_ and extraordinary *n*_*e*_ refractive indices and the measured position of the selective reflection band as follows:


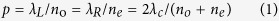


where *λ*_L_ and *λ*_R_ are the short and long wavelength edges and *λ*_c_ is the central wavelength of the selective reflection band. Based on the measured width of the selective reflection band, the refractive indices are taken to be *n*_*o*_ = 1.50, *n*_*e*_ = 1.65 at room temperature and *n*_*o*_ = 1.53, *n*_*e*_ = 1.59 at the high temperature of the experiment.

**Figure 5:**
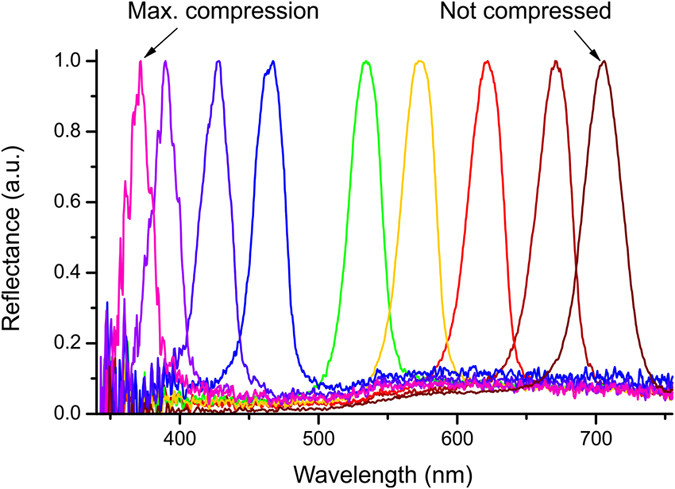
Blue-shift of selective reflection band in CLCE film created by a uniaxial strain along helical axis.

Using the compressing device, we were able to uniformly compress the CLCE film and dramatically shift the selective reflection band. The largest observed blue-shift of the selective reflection band was from λ_c_ = 705 nm to λ_c_ = 370 nm, as shown on Fig. 5. This tuning of the selective reflection band corresponds to a change of helical pitch from 453 nm to 238 nm corresponding to a compression of 47.5% and was observed in a smaller sample with initial area, in the glassy state, of 7.2 mm^2^.

**Figure 6:**
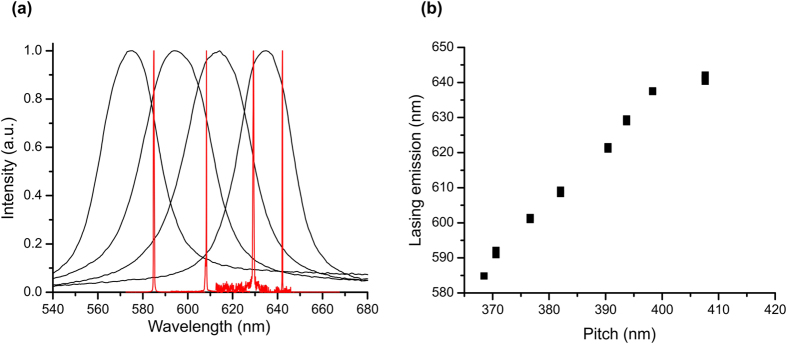
Mechanical wavelength tuning of lasing emission from CLCE film. (**a**) selective reflection band (black) and lasing emission (red) from CLCE film in states with different strain. (**b**) lasing emission wavelength as a function of CLCE helical pitch.

Lasing emission from the CLCE film in a rubbery state at 75 °C was always observed at the low energy edge of the selective reflection band, as shown on Fig. 6(a). Using mechanical tuning of the position of the selective reflection band in the vicinity of fluorescence band of the DCM laser dye, lasing emission was observed from 642.1 nm (λ_c_ = 635 nm) to 584.8 nm (λ_c_ = 584 nm), as shown on Fig. 6 (b). The observed range of tuning of the lasing wavelength is primarily defined by the location of the fluorescence band of selected laser dye, and is not limited by other properties of the CLCE film. The range can therefore be easily extended by using different fluorescent dyes. Since the CLCE film in the rubbery state is contained in a compressing cell located inside of a heating chamber, precise measurements of lasing threshold in the rubbery state are challenging. The compression cell and the chamber of the heating device create losses both of the pump beam and of the emission from the CLCE film which are difficult to quantify. Based on our measurements, we estimate the lasing thresholds from CLCE film in glassy and rubbery state to be approximately the same.

**Figure 7:**
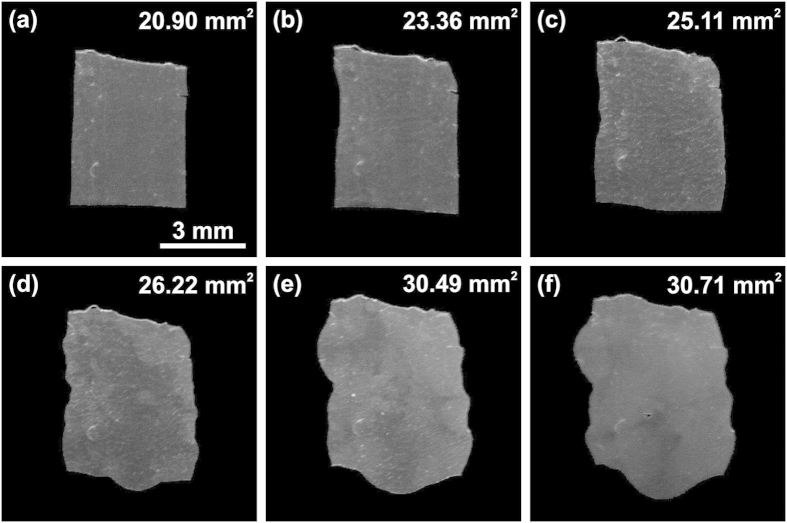
Sequential change of shape and area of the CLCE film under compression.

A complex deformation of the shape of the CLCE film was observed as result of the applied strain. Figure 7 shows a sequential transformation of the shape and area of the CLCE film in the rubbery state. The initial area of the film in a glassy state was 23.64 mm^2^. Regardless of the nontrivial deformation of the shape of the film, the average reflection band remained essentially uniform over the entire film, indicating that the change of shape only shortened the CLC pitch but left the cholesteric helical structure unchanged. In all of the compressed and uncompressed states, the CLCE film was uniformly colored in reflection with local deviation of the CLC pitch in different spots of the film less than 2.4%.

**Figure 8:**
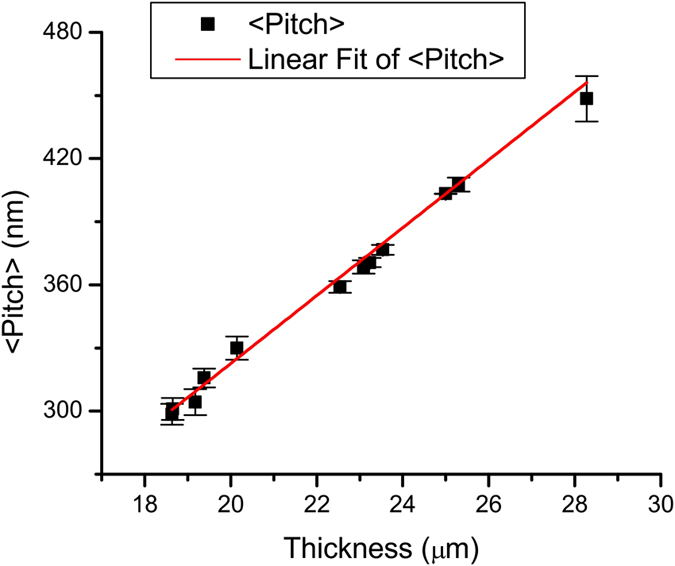
Helical pitch of the CLCE film as a function of film thickness. The error bars show average deviation of the CLC pitch.

We measured the reflectance and area of the CLCE film in a rubbery state and calculated the change of the helical pitch as a function of applied strain. Assuming volume conservation, the thickness of the CLCE film in states of different strain was calculated with reference to the glassy state, where the thickness of the CLCE film is known to be 25 μm. The cholesteric pitch and the area of the CLCE film were found to be linearly dependent on the strain created by the uniaxial compression. Figure 8 shows the change of CLC pitch as a function of the film thickness. Experimental data points fall almost exactly on a line with zero intercept. Upon transition from the glassy to the rubbery state, the CLCE film experienced positive strain corresponding to an increase of the CLC pitch and thickness and a decrease of the area. Uniaxial compression of the film in a rubbery state creates a negative strain corresponding to a decrease of CLC pitch and thickness and an increase of area of the film. The compression of thickness of the CLCE film based on largest measured change of the area of the film was 34.1%.

**Figure 9:**
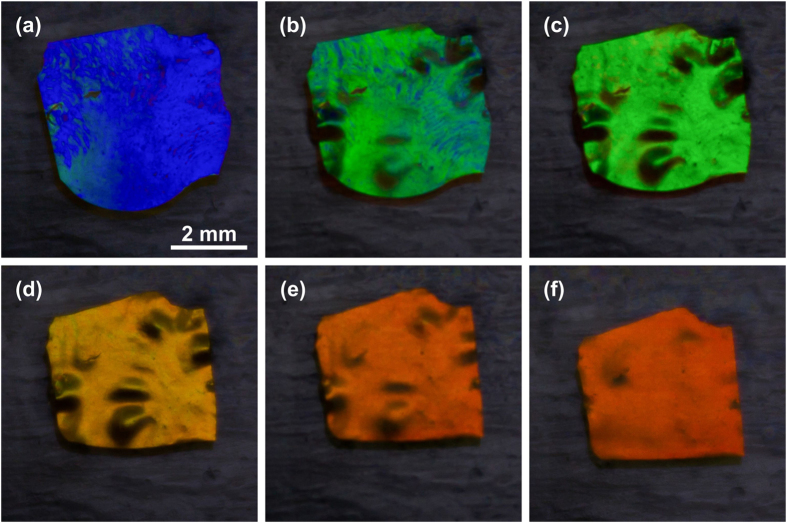
Appearance of the CLCE film in reflected light under white light illumination during free relaxation from compressed to relaxed state with decreasing strain. Photographs taken at the following times: **(a)** 0 s, **(b)** 3 min 36 s, **(c)** 4 min 30 s, **(d)** 7 min 41 s, **(e)** 15 min 11 s, **(f)** 47 min 45 s.

When the applied force causing compression is removed, the CLCE spontaneously returns to its original equilibrium state with film area and selective reflection band matching their original values before the deformation. A sequence of photographs in reflected light of a previously compressed CLCE film during a free recovery process corresponding to a relaxation of the selective reflection band from λ_c_ = ~450 nm to λ_c_ = ~700 nm is shown on Fig. 9. The CLCE film shown on Fig. 9 was compressed in a compression cell, the cell was opened and the film was removed and placed on a glass slide lubricated with silicone oil (viscosity 1 m^2^/s). The glass slide was placed on the surface of a hot stage which is visible on images as a black background. The temperature of the surface of the hot stage was set to 75 °C. The free recovery of the shape and reflectance of the compressed CLCE sample with area of ~15 mm^2^ was completed in ~15 minutes. Films with ~3 times smaller area were able to recover under identical conditions and from similar compression in ~2 minutes, with significantly smaller visible wrinkling.

## Conclusions

We have demonstrated lasing from CLCE films both in glassy and rubbery states. A dramatic reversible blue-shift of the selective reflection band from near IR to near UV ranges of the spectrum was achieved by a novel mechanical compression of the CLCE film using uniaxial strain along the helical axis. Using an accurate measurement scheme based on volume conservation of the CLCE film, we have shown here, for the first time, that both the helical pitch and the wavelength of laser emission from the CLCE film in the rubbery state are linearly dependent on the mechanical strain applied to the film.

## Additional Information

**How to cite this article**: Varanytsia, A. *et al.* Tunable lasing in cholesteric liquid crystal elastomers with accurate measurements of strain. *Sci. Rep.* **5**, 17739; doi: 10.1038/srep17739 (2015).
